# Protective Role of Curcumin against N-Nitrosodiethylamine (NDEA)-Induced Toxicity in Rats

**DOI:** 10.3797/scipharm.1506-06

**Published:** 2015-07-26

**Authors:** Fahad Ali, Rahul, Smita Jyoti, Ambreen Fatima, Saba Khanam, Falaq Naz, Yasir Hasan Siddique

**Affiliations:** Section of Genetics, Department of Zoology, Faculty of Life Sciences, Aligarh Muslim University, Aligarh, Uttar Pradesh, India

**Keywords:** N-Nitrosodiethylamine, NDEA, Curcumin, Oxidative stress markers

## Abstract

The present investigation was aimed at studying the possible role of curcumin against N-nitrosodiethylamine (NDEA)-induced toxicity in albino rats. Administration of NDEA to rats at a concentration of 0.1 mg/ml in drinking water ad libitum for 21 days produced toxicity in them, which was evident from histopathological changes in the rat livers, and increased levels of blood serum enzyme markers, i.e. aspartate transaminase, alanine transaminase, alkaline phosphatase, and lactate dehydrogenase. In addition, the levels of oxidative stress markers like lipid peroxidation (LPO), protein carbonyl (PCC), and glutathione-S-transferase (GST) activity were elevated and the total glutathione (GSH) content was reduced in the livers. The administration of curcumin to rats at concentrations of 10, 20, and 40 mg/ml in drinking water along with 0.1 mg/ml of NDEA for 21 days effectively suppressed NDEA-induced toxicity and also resulted in a dose-dependent reduction in the levels of blood serum enzyme markers (AST, ALT, ALP, and LDH). Moreover, LPO, PCC, and GST activity were reduced and the GSH level was increased upon the administration of curcumin along with NDEA. The results obtained for the comet assay in rat hepatocytes and blood lymphocytes showed a significant dose-dependent decrease in the mean tail length. The micronucleus assay performed on rat hepatocytes also showed a dose-dependent reduction in the frequency of micronucleated cells along with curcumin administration. These results suggest that curcumin has a protective role against NDEA-induced toxicity in albino rats.

## Introduction

There are numerous toxic compounds present in the environment that are capable of inducing biochemical and histological transformation in humans either directly or indirectly [1]. One such compound, N-nitrosodiethylamine (NDEA), belonging to the nitrosamine family is known to be a potent carcinogen [2]. It has been demonstrated that liver cancer can be induced in rats by the oral treatment of NDEA at a concentration of 0.02% for a particular duration [3]. Besides being present in food items like cheese, smoked, salted, and dried fish, cured meat, and alcoholic beverages [4], it is also found in the effluents and smoke released from rubber, dye, metal industries, and cigarettes [4]. The degradation of some therapeutic drugs can also lead to the generation of nitroso compounds [5]. These nitroso compounds are known to cause hepatoportal sclerosis, hepatolobular dilation, fibrosis, tissue necrosis, liver enlargement, pre-neoplastic lesions, and cirrhosis [6, 7]. NDEA is degraded by the action of the cytochrome P450-dependent monooxygenase system to form its active ethyl radicals (CH_3_CH^2+^) [8]. These ethyl radicals and other reactive products soon interact with DNA leading to mutations, elevations in blood serum enzyme markers like aspartate-transaminase (AST), alanine-aminotransferase (ALT), alkaline-phosphatase (ALP), gamma-glutamyl transferase (GGT), lactate-dehydrogenase (LDH), and total bilirubin (TB), increases in oxidative stress markers such as lipid peroxidation (LPO), protein carbonyl content (PCC), superoxide dismutase (SOD), and catalase (CAT), and they also cause neoplastic transformation in liver tissues [9–11]. Thus, to maintain cellular integrity, it is essential to have an effective chemical or reactive oxygen species scavenger system to target both xenobiotic-metabolizing enzymes and free radicals. Several epidemiological and animal studies have provided evidence that high intakes of natural plant products rich in antioxidant properties are associated with the reduction in the progression of various types of cancers [12, 13]. Curcumin, 1,7-Bis(4-hydroxy-3-methoxyphenyl)hepta-1,6-diene-3,5-dione or diferuloylmethane, is a hydrophobic yellow-colored polyphenol extracted from the rhizome of *Curcuma longa* (turmeric). India accounts for more than 90% of the world’s total turmeric production. Thus, it is a common food flavoring and coloring agent in India [14]. Curcumin is the most active component found in turmeric and has been known to exhibit antioxidant, anti-inflammatory, antimicrobial, and anticarcinogenic activities in addition to hepatoprotective and nephroprotective effects [15–19].

Curcumin in rat and human hepatocytes is metabolised into its derivatives such as hexahydrocurcumin, tetrahydrocurcumin, dihydrocurcumin, curcumin glucoronide, hexahydrocurcuminol, and curcumin sulphate [20, 21]. In the present study, the effect of curcumin was studied against NDEA-induced toxicity in Swiss albino rats.

## Experimental

### Chemicals

Curcumin and N-nitrosodiethylamine were purchased from Sigma Chemicals Co. (USA). Triton X, ethidium bromide, agarose (normal and low-melting), dimethylsulphoxide, Tris, EDTA, and all other chemicals were purchased from SISCO Research Laboratories, India. May Grünwald stain and Giemsa stain were procured from Merck Ltd. (India).

### Animals and Treatment

In this study, male Wistar rats weighing 100–120 g were used. The animals were divided into nine groups (5 rats/group). The first group was allowed to feed on N-nitrosodiethylamine (NDEA) dissolved in water (0.1 mg/ml), the second group was allowed to feed on water with NDEA (0.1 mg/ml) plus curcumin (10 mg/ml), the third group was allowed to feed on water with NDEA (0.1 mg/ml) plus curcumin (20 mg/ml), the fourth group was allowed to feed on water with NDEA (0.1 mg/ml) plus curcumin (40 mg/ml), and the fifth group was given normal drinking water which served as the control. The sixth group was taken as a negative control and was allowed to feed on water with dimethysulfoxide (DMSO) (3 µl/ml). The seventh, eighth, and ninth groups were allowed to feed on water with curcumin (dissolved in DMSO) at final concentrations of 10, 20, and 40 mg/ml, respectively. Curcumin was first dissolved in 0.03% DMSO and then in drinking water to make the final concentrations 10, 20, and 40 mg/ml, respectively. The rats were allowed to feed *ad libitum* for 21 days and were sacrificed under mild ether anesthesia. All necessary care was taken while housing and experimenting on the animals. The study was approved by the ethical committee.

### Histological Evaluation of the Liver

A portion of each liver was removed and washed thoroughly with 0.9% saline. The tissue was kept in 10% buffered neutral formalin (BNF) for 24 hrs. Then the fixed liver specimens from each group were embedded in paraffin and processed for light microscopy by staining individual sections with the hematoxylin and eosin stain.

### Biochemical Analysis

The blood samples were collected directly by cardiac puncture in a vacutainer having a clot activator (AKÜret, Medkit). The serum was collected for the biochemical analysis of serum glutamic oxaloacetic transaminase (SGOT), serum glutamic-pyruvic transaminase (SGPT), alkaline phosphatase (ALP), and lactate dehydrogenase (LDH). The levels of the enzymes were estimated according to the method described in the commercial kits (Crest Biosystems, India).

### Preparation of Liver Homogenate

The homogenate was prepared according to the procedure described by Singh *et al*. [22] with a little modification. The livers were washed thoroughly with chilled 0.9% saline. The final wash was given with chilled homogenizing buffer (pH 7.5) containing 0.024 M EDTA, 0.075 M NaCl, and DMSO 10%. After weighing, the liver was mixed, suspended in chilled homogenizing buffer at a concentration of 1 g/ml, and homogenized on ice using homogenization at 1500 rpm. The homogenate was then centrifuged at 7000 rpm for 10 min at 4°C. The supernatant was removed, resuspended in a homogenization buffer, and kept at -20°C for further analyses.

### Estimation of Protein Carbonyl Content

The protein carbonyl content was estimated according to the protocol described by Hawkins *et al*. [23]. The liver homogenate was diluted to a protein concentration of approx 1 mg/ml. About 250 µl of each diluted homogenate was taken in Eppendorf centrifuge tubes separately. To it, 250 µl of 10 mM 2, 4-dinitrophenyl hydrazine (dissolved in 2.5 M HCl) was added, vortexed, and kept in the dark for 20 min. About 125 µl of 50% (w/v) trichloroacetic acid (TCA) was added, mixed thoroughly, and incubated at −20°C for 15 min. The tubes were then centrifuged at 4°C for 10 min at 9000 rpm. The supernatant was discarded and the pellet obtained was washed twice by ice cold ethanol:ethylacetate (1:1). Finally, the pellets were re-dissolved in 1 ml of 6 M guanidine hydrochloride and the absorbance was read at 370 nm.

### Estimation of Lipid Peroxidation

The method described by Siddique *et al*. [24] was used for the estimation of lipid peroxidation in liver cells. Reagent 1 (R1) was prepared by dissolving 0.064 g of 1-methyl-2-phenylindole into 30 ml of acetonitrile. The preparation of 37% HCl served as reagent R2. About 200 µl of diluted liver homogenate (protein concentration of approx 1 mg/ml) was mixed with 300 µl of R1. Then 300 µl of R2 was added to the tube, vortexed, and incubated at 45°C for 40 min. After incubation, the tubes were cooled in ice and centrifuged at 10,000 rpm at 4°C. The absorbance was read at 586 nm.

### Estimation of Glutathione (GSH) Content

The glutathione (GSH) content was estimated colorimetrically using Ellman’s reagent (DTNB) according to the procedure described by Jollow *et al*. [25]. The supernatant was precipitated with 4% sulphosalicyclic acid (4%) in the ratio of 1:1. The samples were kept at 4ºC for 1 hr and then subjected to centrifugation at 5000 rpm for 10 min at 4ºC. The assay mixture consisted of 550 µl of 0.1 M phosphate buffer, 100 µl of supernatant, and 100 µl of DTNB. The OD was read at 412 nm and the results were expressed as µ moles of GSH/gram tissue.

### Estimation of Glutathione-S-Transferase (GST) Activity

The glutathione-S-transferase activity was determined by the method of Habig *et al*. [26]. The reaction mixture consisted of 500 µl of 0.1 M phosphate buffer, 150 µl of 10 mM CDNB, 200 µl of 10 mM reduced glutathione, and 50 µl of supernatant. The OD was taken at 340 nm and the enzyme activity was expressed as µ moles of CDNB conjugates/min/mg/protein.

### Micronucleus Assay

The micronucleus assay was performed according to the method of Igarashi and Shimada [27]. The supernatant was removed and fresh homogenizing buffer was poured to re-suspend the pellet. A drop of the suspension was put at one end of the pre-cleaned, grease-free microscopic slides and was spread using a coverslip held at an angle of 45° into a smooth layer. Before staining, the slides were allowed to air-dry in a dust-free environment for at least 12 hrs. The slides were then stained for 2 min in May-Grünwald stain (0.25% in methanol) followed by staining with 10% Giemsa for 10 min. The slides were then rinsed twice in distilled water, dried, and finally rinsed with methanol. The slides were then cleared in xylene and mounted in DPX. A total of 500 cells were counted per rat for the presence of micronuclei using a light microscope at 40X [28].

### Comet Assay

The comet assay was performed according to the method described by Singh *et al*. [29] and with modification as suggested by Dhawan *et al*. [30]. Frosted microscopic slides were dipped in 1% normal melting agarose and the underside was wiped to remove the agarose (dissolved in PBS). The slides were allowed to dry for 24 hrs. The next day for the liver cells, 40 µl of the cell suspension was mixed with 60 µl of 0.5% low-melting agarose (dissolved in PBS) and was layered on the prepared base slides. For the separation of blood lymphocytes, 20 µl of whole blood in 1 ml of RPMI 1640 and 100 µl of Ficoll histopaque was added and centrifuged at 1500 rpm for 15 min, and finally the obtained pellet was re-suspended in 70 µl of LMPA. For bone marrow, the femurs were perfused with 1 ml of cold homogenization buffer and 10 µl was mixed with 70 µl of LMPA. A cover slip was placed for the even spread of the suspension. The slides were allowed to solidify at 4°C for 10 min. The cover slip was removed and a third layer of 1% low-melting agarose was added and allowed to solidify on an ice pack for 5 min. The slides were then immersed in cold lysis solution (2.5 M NaCl, 100 mM EDTA, 10 mM Tris, and 1% triton X-100, pH 10) at 4°C overnight. The next day, the slides were kept for 30 min in alkaline electrophoretic buffer (300 mM NaOH, 1 mM EDTA; pH > 13) for unwinding of the DNA. Electrophoresis was performed at 4°C at 24 volts for 45 min. The slides were washed with neutralization buffer (0.4 M Tris) and stained with ethidium bromide (20 µg/ml). Three slides were prepared per rat and a total of 50 randomly captured comets per slide under a fluorescence microscope were analyzed for scoring comet tail length by using Comet 1.5 software (TriTek Corporation).

### Statistical Analysis

All data were expressed as the mean ± standard error and Student’s t-test was used for the analysis. Statistical significance was estimated at the 5% level.

## Results and Disscussion

**Fig. 1:**
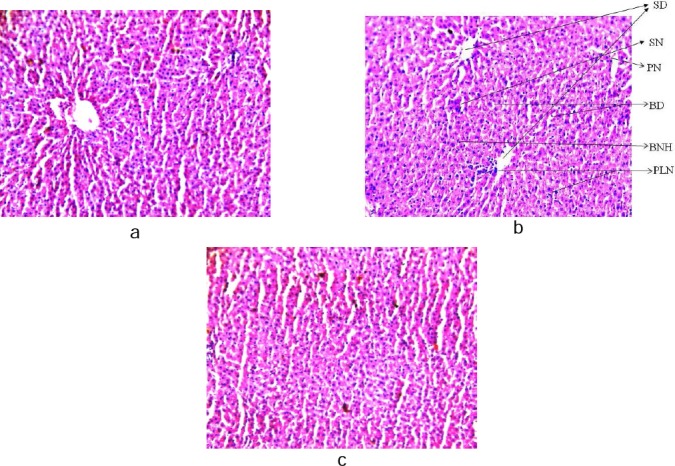
Microscope images of rat livers stained with haematoxylin and eosin (200× magnification) (PLN: partially lysed nuclei; PN: pyknotic nuclei; BD: ballooning degeneration, BNH: binucleated hepatocytes, UNH: uninucleated hepatocytes, SN: swollen nucleus, SD: sinusoidal dilation. (a) Control; (b) Rats exposed to NDEA (0.1 mg/ml) for 21 days; (c) Rats exposed to 0.1 mg/ml of NDEA together with 40 mg/ml of curcumin.

Histopathological study of the liver sections revealed normal structure of the hepatocytes in the control group (Figure 1a). The rats exposed to NDEA (0.1 mg/ml) showed sinusoidal dilation, spaces in tissue architecture, a high frequency of binucleated hepatocytes, pyknotic nuclei, and swollen, empty hepatocytes. Development of small vacuoles in the cytoplasm also gave a histopathological demarcation for fatty change in the liver cells (Figure 1b). A dose-dependent reduction in various deformities like sinusoidal dilation, ballooning degeneration of hepatocytes, swollen and empty hepatocytes, binucleated hepatocytes, partially lysed nuclei, etc. was observed in liver tissue after treatment with curcumin along with NDEA (Figure 1c). The activity of various blood serum enzyme markers in different groups of rats is shown in Table 1. In rats treated with NDEA alone (Group 1), abnormal liver function was found as compared to the control group (Group 5), which was evident by a significant increase in the activities of blood serum enzyme markers such as SGOT, SGPT, ALP, and LDH. However, treatment of animals with NDEA along with different doses of curcumin (Groups 2, 3, and 4) resulted in a significant reduction in SGOT, SGPT, ALP, and LDH levels dose-dependently, with respect to the positive control group (Table 1). There was no significant difference in the values of blood serum enzyme markers in the negative control group (Group 6) and rats treated with different doses of curcumin alone (Groups 7, 8, and 9) as compared to the untreated group (Table 1).

**Tab. 1:**
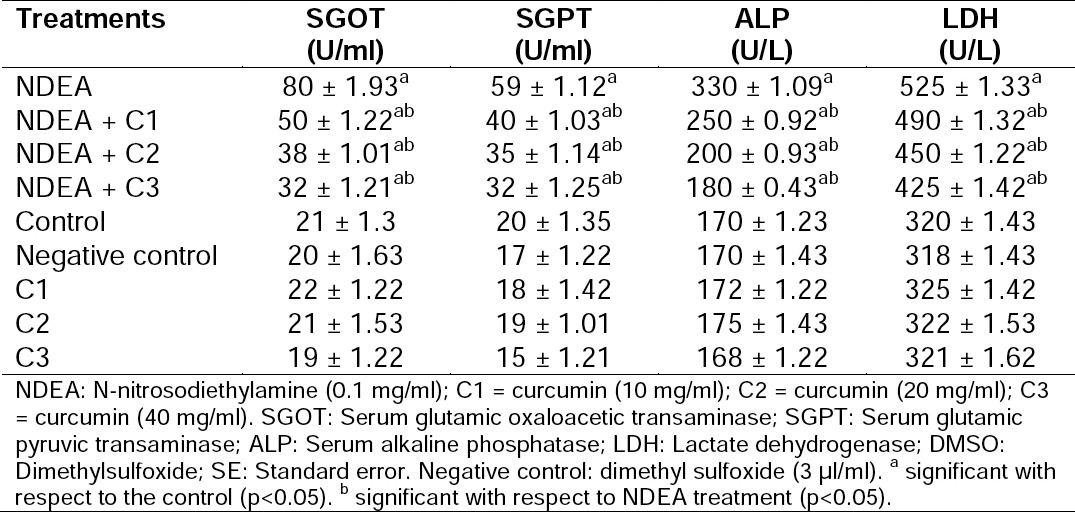
The activity of blood serum enzyme markers in different groups of rats exposed to NDEA alone and together with different amounts of curcumin [mean ± SE]

**Fig. 2:**
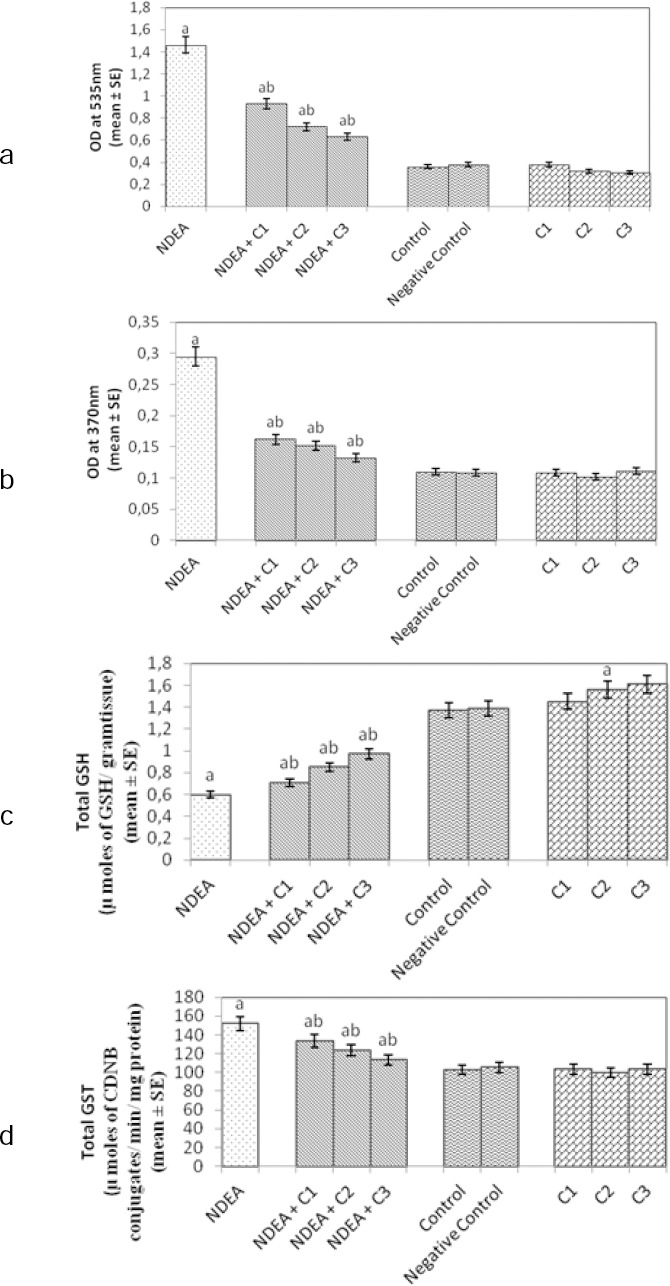
(a) Lipid peroxidation (LPO); (b) Protein carbonyl content (PC); (c) Glutathione reductase (GSH); (d) and Glutathione-S-transferase (GST) measured in rat liver homogenate after 21 days of treatment of rats with NDEA alone, or together with different amounts of curcumin. NDEA: N-nitrosodiethylamine; C1 = curcumin (10 mg/ml); C2 = curcumin (20 mg/ml); C3 = curcumin (40 mg/ml); negative control: dimethyl sulfoxide (3 µl/ml); SE: standard error. ^a^Significant compared with the control (p < 0.05); ^b^significant compared with NDEA treatment (p < 0.05).

Figures 2 a, b, c, and d show the effect on oxidative stress markers in the control and experimental group of rats. In NDEA-treated rats (Group 1), the levels of LPO, PC content, and GST were significantly increased while the level of GSH was significantly reduced with respect to the control (Group 5). Different doses of curcumin, when administered to the rats along with NDEA (Groups 2, 3, and 4), showed significant decreases in levels of LPO, PC content, and GST activity, while a significant increase in levels of GSH was observed when compared to the positive control group (Group 1). Oxidative stress markers examined from the group of rats treated with various doses of curcumin alone showed no significant difference with respect to the control (Groups 7, 8, and 9).

**Fig. 3:**
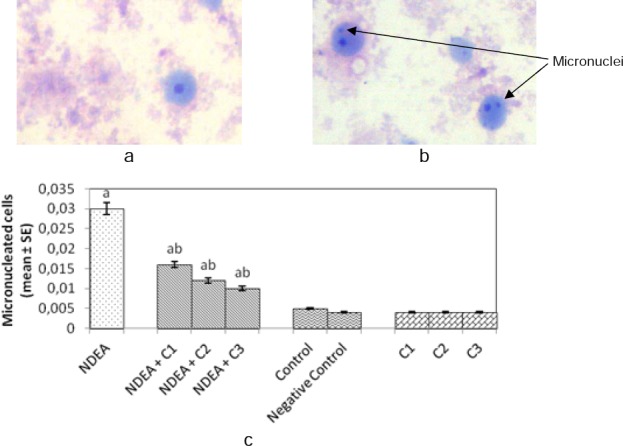
(a) Normal hepatocyte from the control group; (b) Micronucleated hepatocytes from the positive control group; (c) Frequency of micronucleated cells in rat hepatocytes, after 21 days of treatment of rats with N-nitrosodiethylamine alone and together with different amounts of curcumin. NDEA: N-nitrosodiethylamine; C1 = curcumin (10 mg/ml); C2 = curcumin (20 mg/ml); C3 = curcumin (40 mg/ml); negative control: dimethylsulfoxide (3 µl/ml); SE: standard error. ^a^Significant compared with the control (p < 0.05); ^b^significant compared with NDEA treatment (p < 0.05).

Figures 3a and b showed normal hepatocytes in the control group (Group 5) with prominent, healthy nuclei and micronucleated hepatocytes from the NDEA-treated group of rats (Group 1), respectively. The frequency of micronucleated hepatocytes in different experimental groups of rats is shown in Fig. 3c. The results indicate that there was a significant increase in the frequency of micronucleated hepatocytes in rats treated with NDEA alone (Group 1) when compared to the control group (Group 5). However, a dose-dependent decrease was observed with the frequency of micronucleated hepatocytes in the groups treated with NDEA along with different doses of curcumin (Groups 2, 3, and 4). Moreover, the negative control group (Group 6) and groups with different doses of curcumin alone (Groups 7, 8, and 9) showed no significant difference in the frequency of micronucleated hepatocytes as compared to the control group (Group 5).

**Fig. 4:**
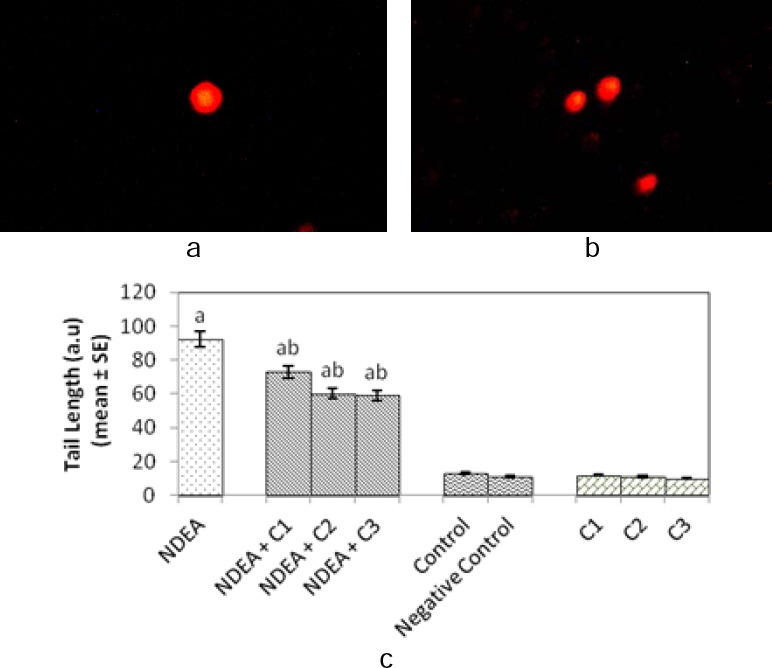
Comet assay performed with rat hepatocytes: (a) Control; (b) After 21 days of treatment of rats with N-nitrosodiethylamine alone (0.1 mg/ml); (c) Comet tail length in normal rat hepatocytes and after 21 days of treatment of rats with N-nitrosodiethylamine alone (0.1 mg/ml) and together with different doses of curcumin. NDEA: N-nitrosodiethylamine; C1 = curcumin (10 mg/ml); C2 = curcumin (20 mg/ml); C3 = curcumin (40 mg/ml); negative control: dimethyl sulfoxide (3 µl/ml); SE: standard error. ^a^Significant compared with the control (p < 0.05); ^b^significant compared with NDEA treatment (p < 0.05).

**Fig. 5:**
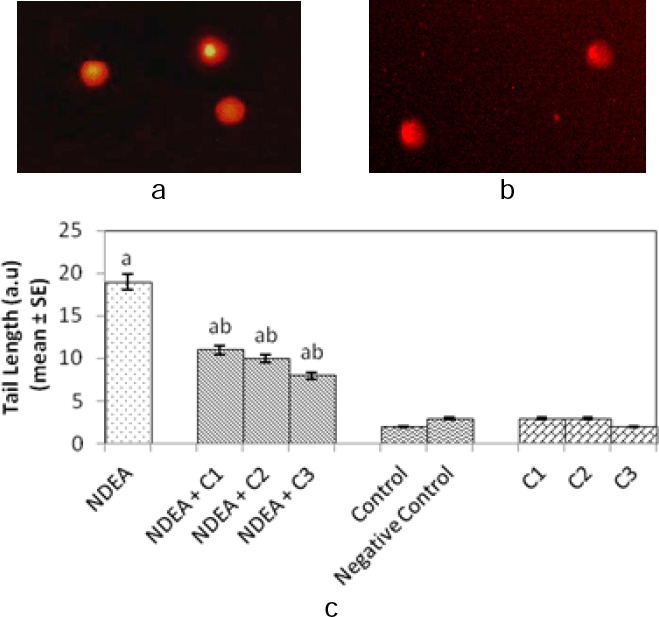
Comet assay performed with rat blood lymphocytes: (a) Control; (b) After 21 days of treatment of rats with N-nitrosodiethylamine alone (0.1 mg/ml); (c) Comet tail length in normal rat blood lymphocytes and after 21 days of treatment of rats with N-nitrosodiethylamine alone (0.1 mg/ml) and together with different doses of curcumin. NDEA: N-nitrosodiethylamine; C1 = curcumin (10 mg/ml); C2 = curcumin (20 mg/ml); C3 = curcumin (40 mg/ml); negative control: dimethyl sulfoxide (3 µl/ml); SE: standard error. ^a^Significant compared with the control (p < 0.05); ^b^significant compared with NDEA treatment (p < 0.05).

The comet assay performed on rat hepatocytes and blood lymphocytes shown in Fig. 4a, 4b and 5a, 5b, respectively, revealed that there was more DNA damage in the positive control group (Group 1) with respect to the control group (Group 5), which is evident from the longer mean tail length of comets in the positive control group (Figures 4c and 5c). The groups treated with NDEA along with different amounts of curcumin (Groups 2, 3, and 4) showed a significant and dose-dependent decrease in the mean tail length compared to the positive control group (Figures 4c and 5c). There was no significant difference in the mean tail length of the control (Group 5), negative control (Group 6), and group of rats (Groups 7, 8, and 9) treated with different doses of curcumin alone (Figures 4c and 5c).

The results of the present study reveal that curcumin is potent enough to reduce the toxic effect of NDEA in rats. Rats exposed to 0.1 mg/ml of NDEA for 21 days induced hepatotoxicity, which was clear from the increase in the levels of SGOT, SGPT, ALP, and LDH. A dose-dependent reduction in the toxicity was observed in rats exposed to different doses of curcumin along with 0.1 mg/ml of NDEA. Reduction in the liver damage and toxicity was confirmed from a decrease in the levels of SGOT, SGPT, ALP, and LDH. These observations were correlated with the liver damage induced by NDEA, disrupting liver cell metabolism and membrane stability and subsequently, causing distinctive changes in the liver marker enzymes that enter the circulation [10, 31]. There is considerable support to the findings that NDEA induces oxidative stress and cellular injury due to the involvement of ROS [32–34]. An increase in ROS production significantly increases the levels of liver enzyme markers and oxidative stress [35]. The ability of curcumin to reduce these serum enzymes may be attributed to its free radical-scavenging property and it can induce arachidonic acid metabolism via the COX and LOX pathway [36, 37]. Reduction in GST and GSH activity upon treatment of rats with curcumin is concurrent with the studies of other workers [38, 39]. The increases in oxidative stress markers like lipid peroxidation and protein carbonyl content in liver cells after treatment with NDEA have also been reported earlier [8]. The curcumin treatment results in a dose-dependent reduction in lipid peroxidation in the liver. This may be due to the ROS scavenging potential of curcumin and modulation of antioxidant enzymes in the hepatocytes [40, 41].

Reports suggest that curcumin showed no toxicity and no side effects when taken as a medicine at a dose concentration as high as 8000 mg/day for 3 months [42]. Others suggest a maximum dose limit for curcumin to be 3600 mg/day for 7 weeks [43, 44]. Curcumin administration to NDEA-treated rats has resulted in healthy, recoverable changes in liver histology. The systemic administration of curcumin to rats bearing highly cathetic Yoshida AH-130 ascites hepatoma resulted in a significant inhibition (31%) of tumor growth [45]. In addition to this, curcumin has also shown a protective effect over lindane-induced oxidative damage and results in histological alterations [46]. There are numerous carcinogens and mutagens known to cause mutations and cell death by altering the structure of DNA. Among many genotoxic assays, the micronucleus (MN) assay and comet assay are commonly performed to assess the chromosomal damage *in vitro* and *in vivo* caused by various mutagens [47]. In this study, the MN assay was performed on liver cells of treated and control rats. The results obtained clearly suggest the protective nature of curcumin against NDEA. Curcumin doses alone did not induce MN formation at a significant level compared to the control. Our data regarding the comet assay on blood lymphocytes and hepatocytes also suggest the anti-genotoxic nature of curcumin towards NDEA-induced genotoxicity. The curcumin treatment showed a dose-related reduction in DNA damage in hepatocytes and blood lymphocytes as is evident from a decrease in mean comet tail length. Metabolic transformation of NDEA to its active ethyl radical metabolites (CH_3_CH^2+^) and other free radicals by the P-450-dependent monooxygenase system is responsible for the DNA damage in cells [33]. Further, the detoxification of these NDEA byproducts takes place by phase II enzymes like GST and GSH. The study showed an increase in GST and a decrease in GSH activity in NDEA-treated rats. A dose-dependent inverse relation in the activity of both these enzymes was observed when different concentrations of curcumin along with NDEA was administered to rats.

The systemic bioavailability of curcumin is very poor, thus pharmacological activity may be mediated in part by its metabolites Lin *et al*. [48] and show that curcumin is first biotransformed to dihydrocurcumin and tetrahydrocurcumin. The latter compounds are reduced to form hexahydro-curcumin. Conjugation of these intermediates with glucoronides produces dihydrocurcumin glucoronide, tetrahydrocurcumin glucoronide, and hexahydro-curcumin glucoronide [20, 21]. After pre-oral administration of 500 mg/kg of curcumin, the parent drug was present in plasma at levels near the detection limits. The major products of curcumin biotransformation identified in rat plasma were curcumin glucoronide and curcumin sulphate, whereas hexahydro-curcumin, hexahydro-curcuminal, and hexahydrocurcuminal glucoronide were present in small amounts [20]. As far as the structural activity of curcumin and its derivatives are concerned, the phenolic analogs are more active than the non-phenolic analogs [49]. It has been showed that the presence of hydroxyl groups at the *ortho* position on the aromatic rings and the beta-diketone functionality were required for high potency in inducing phase 2 detoxification enzymes [50, 51]. The antioxidant activity increases when methyl groups are added to the *ortho* position of the phenolic group. The phenolic group is essential for the free radical scavenging activity and the presence of a methoxy group further enhances the activity. Moreover, the presence of more than one hydroxyl group as in the curcumin derivative bis(3,4-dihydroxy-cinnamoyl)methane confers better activity than curcumin itself [52]. Thus, most biological effects of curcumin *in vitro* as well as *in vivo* are due to its antioxidant effects and its role in scavenging free radicals [49, 53].

## Conclusion

The results from the present study clearly demonstrate that curcumin possesses antioxidant and anti-genotoxic properties and could serve as a free radical scavenger. It provides protection to rats against hepatotoxicity induced through the administration of NDEA.
